# Five-Year Weight Loss Experience of Outpatients Receiving Laparoscopic Adjustable Gastric Band Surgery

**DOI:** 10.1007/s11695-013-0881-7

**Published:** 2013-02-28

**Authors:** Chris Cobourn, Mary Ann Chapman, Arlene Ali, John Amrhein

**Affiliations:** 1 Surgical Weight Loss Centre, 1413 Hurontario St., Mississauga, Ontario L5G 3H5 Canada; 2 Visage Communications, Mead, WA USA; 3 Allergan, Inc., Markham, Ontario Canada; 4 McDougall Scientific, Ltd., Toronto, Ontario Canada

**Keywords:** Laparoscopic adjustable gastric banding (LAGB), Obesity, Lap-Band, Outpatient

## Abstract

**Background:**

This study evaluated the efficacy and safety of laparoscopic adjustable gastric banding (LAGB) in a large cohort of morbidly obese patients followed for up to 5 years.

**Methods:**

Morbidly obese patients, ≥16 years of age, who underwent LAGB surgery at the Surgical Weight Loss Clinic in Ontario, Canada, between May 2005 and January 2011 were eligible for this retrospective chart review. Electronic files were searched to identify all patients who met the inclusion/exclusion criteria. Demographics, weights at baseline and follow-up visits (up to 60 months following surgery), and post-operative complications were documented. As follow-up visits occurred at unevenly spaced intervals within and across patients, modeling methods were used to more accurately assess mean % weight loss (WL) and % excess weight loss (EWL) over time.

**Results:**

This study included 2,815 patients (82 % female, mean age 43 years, mean baseline BMI 44.6 kg/m^2^) followed for a mean of 21.8 ± 15.4 months. Complications developed in 238 patients (8.5 %), the most frequent being prolapse/slippage (4.2 %), tubing/access port problems (1.2 %), and explantation (1.2 %). Mean %WL and %EWL progressed continuously over the first 2.5 years post-LAGB, plateauing at 20 and 49 %, respectively, for up to 5 years of follow up. Factors associated with increased weight loss were time since surgery, greater baseline weight (excess weight), older age at time of surgery, and male gender.

**Conclusions:**

Weight loss was maintained for up to 5 years in our population of patients who underwent LAGB for the treatment of morbid obesity.

## Introduction

Morbid obesity exacts an immense toll on personal health, increasing the risk of type 2 diabetes, ischemic heart disease, stroke, hypertension, obstructive sleep apnea, degenerative joint conditions, and multiple cancers. [1, 2] Conservative weight management techniques, including medications, often fail to achieve substantial and prolonged weight loss in these individuals, hence the increasing popularity of bariatric surgery.

Laparoscopic adjustable gastric band (LAGB) surgery is a well-established bariatric procedure that results in substantial and durable weight loss as well as a significant decrease in obesity-related comorbidities. [3, 4] Compared with other bariatric procedures, LAGB is associated with lower rates of complications, shorter hospital stays, lower hospital readmission rates, and lower mortality rates, and is reversible. [2, 5–7] LAGB can be routinely performed in an outpatient setting due to the predictable and reproducible nature of the laparoscopic procedure. [8–11]

An important consideration with any obesity treatment is the durability of the weight loss that is achieved. [12] A number of recent publications have documented medium- to long-term maintenance of significant weight loss with LAGB. [13–19] The present study adds to that literature by evaluating the efficacy and safety of LAGB in a large cohort of morbidly obese patients or obese patients with at least one comorbidity who underwent surgery in an outpatient Canadian center and were followed for up to 5 years.

## Materials and Methods

### Study Design, Subjects, and Surgeries

This retrospective chart review included patients who underwent LAGB surgery at the Surgical Weight Loss Clinic (SWLC) in Mississauga, Ontario, Canada, between May 2005 and January 2011. To be included in the analysis, subjects must have had a reliable start date for weight, been 16 years of age or older, and had a body mass index (BMI) ≥35 kg/m^2^ or a BMI ≥30 and <35 kg/m^2^ with at least one associated comorbidity. Patients who had previously undergone bariatric surgery, including those who underwent LAGB previously through a different surgical center, were excluded.

The standard pre-operative protocol at SWLC included a very low calorie diet (VLCD) product (Optifast® or equivalent) for at least 2 weeks prior to surgery in order to reduce fatty infiltration of the liver. [20, 21] The exact duration of VLCD varied with the patient's weight at the time of the surgical consultation.

All LAGB surgeries were performed by one of the authors (CC) or one of the other surgeons at SWLC using LAP-BAND® Adjustable Gastric Band (Allergan, Inc., Irvine, CA, USA) using the standardized pars flaccida technique as described previously. [11] Allergan 10 cm and VG bands were used prior to the introduction of the AP System® which was used beginning in November 2006. Hiatal hernias and crural defects were repaired whenever a defect in the crura was identified.

Patients were followed as per the usual procedures at SWLC, which includes counseling and band adjustments as needed. There was no rigid schedule, but generally follow-ups were monthly for the first 3 months, bi-monthly through the end of the first year, every 6 months for the second year, then annually or more frequently as needed. Patients were encouraged to follow up whenever weight loss slowed, satiety was not present with reduced portions of food, or if there were symptoms that were unanticipated.

### Electronic Database and Outcome Measures

Electronic files are kept for all patients treated at SWLC, and these files were searched to identify those who met the inclusion/exclusion criteria. Baseline demographic data including age and sex (male/female) were collected for these individuals, as well as weights at baseline and at each visit for up to 60 months following surgery, as available. Given the retrospective nature of this study, patients were not followed up at pre-defined intervals but rather at intervals based on patient progress as described earlier.

The main outcome measures were percentage weight loss (WL) and excess weight loss (EWL). Percentage excess weight loss (%EWL) was calculated as weight loss divided by excess weight at baseline, with the quotient multiplied by 100: %EWL = (BMI at baseline−BMI at follow-up)/(BMI at baseline−ideal BMI) * 100, where ideal BMI is assumed to be 25 kg/m^2^. BMI was calculated as weight in kg/height in m^2^.

As part of the usual protocol at SWLC, all known complications are recorded in our medical records. There were no peri-operative deaths in this series. We have previously published a detailed report of the short-term complications of LAGB at our center [11].

### Statistical Analysis

Follow-up visits occurred at unevenly spaced intervals within and across patients; therefore, it was not appropriate to calculate simple arithmetic means at specific time points. To account for this uneven spacing of weight measurements, a random coefficient modeling method [22] was used to more accurately evaluate the mean weight loss and excess weight loss over time. The 2,815 patients were divided into two data sets of about equal size. A random sample of one half of the patients, stratified by gender, was selected to generate a data set that was used to define the model. The remaining patients were set aside and used to validate the final model.

Data exploration revealed a weight loss pattern that increased in a curvilinear pattern for approximately 2.5 years following implantation, at which time a plateau was reached. Therefore, a nonlinear random coefficients model was fit using the NLMIXED Procedure of SAS/STAT version 9.2. [14] Models for weight loss and excess weight loss were fit separately. Linear and quadratic terms for time (days after implantation) were included in the model. We also examined the effects of selected variables on weight loss: sex, baseline age, baseline BMI, baseline weight, and baseline excess weight. These variables and their interactions were tested as covariates in the model. Akaike's information criterion (AIC) and statistical significance (alpha = 0.05) were used to judge the form of the final models. Because mixed modeling methods use all available data, no imputation for “missing” measurements was necessary.

Complications and adverse events were summarized with frequencies and percentages using the entire cohort of patients. All calculations and analyses were performed using SAS/STAT version 9.2.

## Results

A total of 2,815 patients met the inclusion/exclusion criteria and were included in this study. Subject demographics are shown in Table 1. Most patients were female (82 %), with a mean age of 43 years and a mean baseline BMI of 44.6 kg/m^2^ (range 27.3 to 102.7 kg/m^2^). All patients considered for surgery had a BMI ≥35 kg/m^2^ or a BMI ≥30 and <35 kg/m^2^ with at least one associated comorbidity at the time of consultation, but may have lost weight with the VLCD as previously described, and thus had BMI <30 at the time of surgery.

**Table 1:** Patient characteristics and follow-up

Characteristic	*N* = 2,815
Age (years), mean (SD)	43.3 (10.9)
Gender—female, *n* (%)	2,313 (82.2 %)
Weight (kg), mean (SD)	124.8 (27.7)
BMI (kg/m^2^), mean (SD)	44.6 (8.3)
Baseline weights (kg) by age	
<35 years (*n* = 627)	129.2 (31.0)
35 to 50 years (*n* = 1,416)	124.3 (27.1)
>50 years (*n* = 772)	122.2 (25.2)
Follow up visits^a^	
Time of surgery	*n* = 2,764
3–9 months	*n* = 2,281
>9-18 months	*n* = 1,772
>18-30 months	*n* = 1,354
>30-42 months	*n* = 719
>42-54 months	*n* = 304
>54-65 months	*n* = 74

^a^Post-surgery visits are subsequently referred to as 6 months and 1–5 years for ease of reference

Patients were followed for a mean of 653.5 days (21.8 months). The numbers of patients for whom weight loss data were available over time are shown in Table 1. On average, patients received 6.2 band adjustments in the first year and 3.4 adjustments in the second year. Overall, 533 of 2,815 (19 %) patients were defined as lost to follow-up (LFU; i.e., no recorded visit for at least 18 months prior to the closure date of the study).

### Weight Loss

**Fig. 1:**
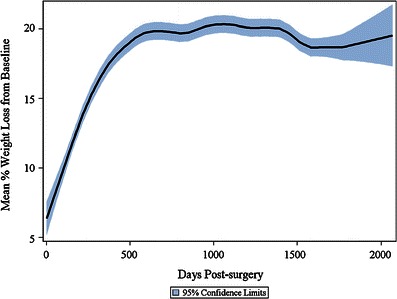
Mean percentage weight loss from pre-surgery baseline. Graph shows the mean ±95 % confidence intervals (*shaded*) for the overall study population

**Fig. 2:**
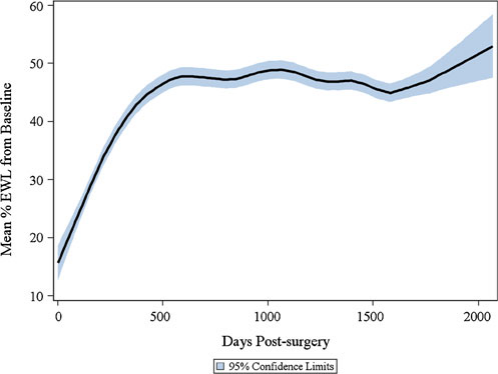
Mean percentage EWL from pre-surgery baseline. Graph shows the mean ±95 % confidence intervals (*shaded*) for the overall study population

Both WL and EWL progressed continuously over the first 30 months post-surgery and were maintained for 5 years of follow-up (Figs. 1 and 2). Mean percent WL and percent EWL plateaued at 20 and 49 %, respectively, post-LAGB. Time since surgery was the dominant factor determining the amount of weight loss, with WL and EWL increasing over time post-surgery until the plateau was reached at 2.5 years post-LAGB for %WL (95 % CI 2.46–2.51) and 2.5 years post-LAGB for %EWL (95 % CI 2.43–2.48).

**Fig. 3:**
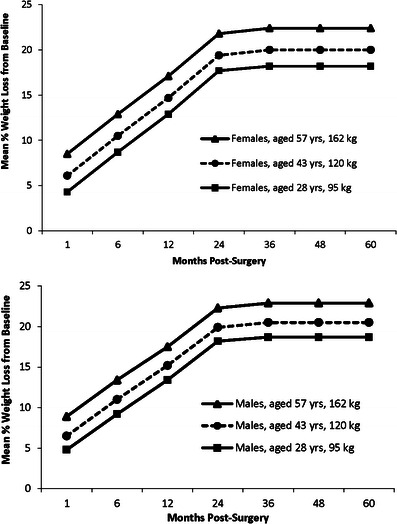
Mean percentage weight loss from pre-surgery baseline showing the effects of age, baseline weight, and sex. Baseline ages of 28, 43, and 57 years and weights of 95, 120, and 162 kg are the 10, 50, and 90 quintiles, respectively, of the whole study population. Graphs show the mean % weight loss for females and males in the 50 % age and baseline weight quintiles (43 years, 120 kg) and the two groups showing the largest differences in percentage weight loss at all time points

**Fig. 4:**
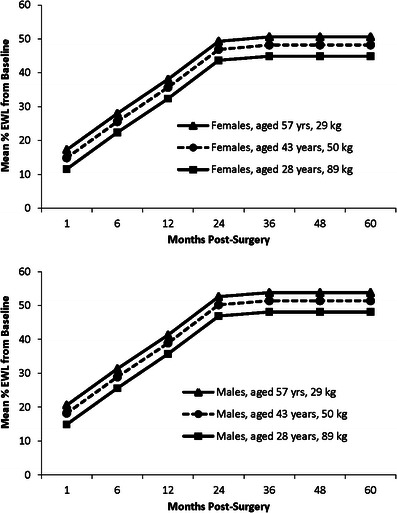
Mean percentage excess weight loss from pre-surgery baseline showing the effects of age, baseline weight, and sex. Baseline ages of 28, 43, and 57 years and excess weights of 29, 50, and 89 kg are the 10, 50, and 90 % quintiles, respectively, of the whole study population. Graphs show the mean % excess weight loss for females and males in the 50 % age and baseline excess weight quintiles (43 years, +50 kg) and the two groups showing the largest differences in percentage weight loss at all time points

The next most important variables associated with weight loss were baseline weight (excess weight), age, and sex (Figs. 3 and 4). Higher weight at baseline, older age, and male sex were associated with greater %WL. Older age and male sex were also associated with greater %EWL, but patients with higher weights at baseline actually lost a lower % of excess weight than those with lower baseline weights. Specifically, patients with higher baseline weights lost 0.04 % more weight per kilogram, but 0.05 % less excess weight per kilogram, than those with lower baseline weights. Older patients realized 0.05 % higher WL and 0.1 % higher EWL for each year of increased age. Comparing males and females of equal age and baseline weight, females experienced 0.5 % lower WL than males (not statistically significant) and 3.2 % lower EWL (*p* < 0.0001).

The level at which weight loss plateaued for each individual patient depended on the patient's age, sex, and baseline weight (excess weight). Based on these factors, the predicted weight loss for an individual patient could be calculated by the following equations:

$$ \%\mathrm{WL}=13.5+0.05\times \mathrm{age}+0.04\times \mathrm{baseline}\ \mathrm{weight}-0.5\times \mathrm{sex} $$

$$ \%\mathrm{EWL}=50.2+0.10\times \mathrm{age}-0.05\times \mathrm{baseline}\ \mathrm{excess}\ \mathrm{weight}-3.2\times \mathrm{sex} $$

where age is expressed in years, weight and excess weight are in kilograms, and sex is classified as 0 (male) or 1 (female).

For example, based on the weight loss outcomes of our patient cohort, a 43-year-old male with a baseline weight of 119 kg and a baseline excess weight of 50 kg would be expected to reach a plateau of 20.5 % weight loss and 51.9 % excess weight loss at 2.5 years post-surgery. A 43-year-old female with the same baseline weight and excess weight would reach a plateau of 20.0 % weight loss and 48.7 % excess weight loss (Figs. 3 and 4).

### Model Validation

Model assessment showed that the assumption of normality was satisfied. The %WL and %EWL models fit the data set extremely well, meeting the criteria for judging acceptability of the final models. When tested using the validation data, the models performed well, with the mean absolute error (MAE) in %WL ranging from 2.4 to 11.1 % over time. Results for percent EWL showed similar trends. MAE for %EWL is higher than for %WL due to the increased possible range of values (i.e., patients can lose more than 100 % of their excess weight, but not 100 % of their weight).

### Complications/Adverse Events

A total of 238 (8.5 %) patients experienced 260 adverse events (Table 2). The most frequent complications, each of which occurred in fewer than 5 % of patients, were proximal pouch dilatation (PPD, prolapse/slippage) (*n* = 118, 4.2 %), tubing/access port problems (*n* = 35, 1.2 %), explantation (*n* = 35, 1.2 %), and erosion into the gastric lumen (*n* = 14, 0.5 %). None of these complications was severe according to the Parikh classification [5] or life-threatening. All adverse events resolved with repositioning, replacement, or removal of the band or adjustment port. Further analysis of these adverse events is the subject of a separate publication that is currently in preparation. Nine patients died during the course of follow-up. Three of the deaths were due to malignancies, three to myocardial infarction, one to cerebral aneurysm, one apparent suicide, and one unknown cause.

**Table 2:** Adverse events

Adverse event	Number (%) of patients(*N* = 2,815)	Number of events
Any	238 (8.5 %)	260
Prolapse/slippage	118 (4.2 %)	119
Tubing/access port problems	35 (1.2 %)	39
Explantation	35 (1.2 %)	35
Erosion into gastric lumen	14 (0.5 %)	14
Death	9 (0.3 %)	9
Wound/minor port site infection	8 (0.3 %)	8
Access port site infection	4 (0.1 %)	4
Intraabdominal bleeding	2 (0.1 %)	2
Deep venous thrombosis	2 (0.1 %)	2
Hernia port site	1 (0.0 %)	1
Intraoperative needle lost	1 (0.0 %)	1
Respiratory insufficiency	1 (0.0 %)	1
Small bowel obstruction	1 (0.0 %)	1
Other	24 (0.9 %)	24

## Discussion

The main finding of this study was that weight loss was maintained for up to 5 years in our population of patients who underwent LAGB for the treatment of morbid obesity. On average, patients achieved nearly 50 % EWL by 2.5 years post-surgery, which remained steady in our population followed for up to 3 (*n* = 719), 4 (*n* = 304), and 5 years (*n* = 74). The amount of EWL documented in the present study agrees closely with the findings from a review of 35 studies of diabetic patients who underwent LAGB, where weight loss was found to progress over the first 2 years post-surgery to reach a mean of 47 % EWL at 2 years. [23]

The maintenance of weight loss with LAGB found in the present study is also in line with findings from a number of other publications. In an Austrian study of 276 patients, mean EWL was maintained at more than 65 % for 10 years following LAGB surgery. [16] A French study of 140 patients showed an increase in EWL from 1 to 5 years following LAGB, for a mean of 46 % EWL at the latter time point. [13] Similar results have been reported by other groups and in several meta-analyses, as summarized in Table 3, although positive long-term results are not universal. [24, 25]

**Table 3:** Summary of % excess weight loss with LAGB in the published literature

Study	Number of patients in study population/follow-up time point(s)	Mean % excess weight loss	Follow-up duration
Spivak [25]	148/127, ≥ 5 years	46	7 years
Alhamdani [40]	575/312, 2 years/66, ≥ 5 years	40	≥5 years
Lanthaler [16]	276/221 (estimated from 80 % follow up)	64	10 years
Caiazzo [13]	143/140	46	5 years
Himpens [15]	151/82	43	13 years (median)
Ray [17]	442/135, 3 years/31, 5 years	51	3 years
		60	5 years
Garb [14]	Meta-analysis of 28 studies	43	1 year (15 studies)
		50	2 years (12 studies)
		55	3 years (9 studies)
O'Brien [3]	Meta-analysis of 18 studies; 4,456, 1 year/3,383, 2 years/640, 5 years	42	1 year (11 studies)
		53	2 years (11 studies)
		55	5 years (5 studies)

A number of studies have found that initial loss of excess weight is greater with gastric bypass than LAGB. [3, 12, 14, 26, 27] However, an analysis of pooled data from 18 gastric bypass and 18 LAGB studies found that the total EWL over time was not different between the two procedures at the later follow-up time points (gastric bypass vs. LAGB: 62 vs. 55 % at 3 years, 58 vs. 55 % at 5 years, and 55 vs. 51 % at 7 years).[3] This suggests that weight loss is more gradual with LAGB than gastric bypass but that it is just as durable and of similar magnitude (i.e., weight loss efficacy) over the long term. One study reported EWL at 7 years to be 58.6 % with gastric bypass and 46.3 % with LAGB in matched cohorts of >100 patients per group.[25] The 48 % EWL with LAGB observed in this study is in line with the literature showing that patients lose a mean of 43 to 64 % of excess weight over the long term (Table 3). In the absence of randomization, it is not possible to conclude that the apparent differences between LAGB and gastric bypass reported in the matched cohort study [25] are due to the procedures themselves (i.e., patient selection factors on which groups were not matched could have contributed to the differential weight loss).

Many studies have shown that resolution of obesity-related comorbidities depends on significant and sustained weight loss and excess weight loss [28–30]. Results of a controlled study found that, among individuals who have had diabetes for less than 2 years, the disease remits in most patients who lose at least 10 % of their body weight following LAGB, whereas the disease does not remit in most patients who lose less than 10 % of their body weight following conventional therapy [28]. Two recent prospective studies exploring mechanisms of type 2 diabetes remission have demonstrated that early improvements of insulin sensitivity and intracellular glucose disposition were secondary to caloric restriction shortly after surgery and from the amount of weight lost over time.[31, 32] This suggests that the predominant effect of bariatric surgery on type 2 diabetes is due to weight loss, despite changes in gut hormones. Remission of diabetes was more likely to be observed in patients with a shorter history and better control of type 2 diabetes prior to bariatric surgery.[31, 33, 34] A recent longitudinal study found that diabetes duration <4 years, body mass >35 kg/m^2^, and fasting C-peptide >2.9 ng/mL were pre-operative factors predicting remission of diabetes at 1 year after gastric bypass.[35] Taken together, these studies suggest that, in order to achieve remission of disease, surgical intervention should be considered at an early phase of diabetes in the obese diabetic patient.

Both LAGB and gastric bypass reduce body weight by approximately 20–40 % and excess body weight by approximately 50–75 % over the long term.[3, 35, 36] Although weight loss at 1 to 2 years post-operatively is more rapid following Roux-en-Y gastric bypass (RYGB) or laparoscopic sleeve gastrectomy (LSG), excess weight loss for LAGB and LSG over time is similar (50–60 %) and somewhat lower than that reported for RYGB (60–75 %); however, morbidity at 1 year is lower for LAGB (5 %) compared to RYGB and sleeve gastrectomy (11–15 %).[37] Given that gastric bypass, sleeve gastrectomy, and LAGB surgeries are all associated with clinically meaningful weight loss, the choice of bariatric surgery should involve careful consideration of both surgical and patient factors. Surgical factors include the expertise and experience of the treatment center, the risk of surgical complications, the complexity and reversibility of the procedure, and the availability of aftercare.[37] In addition, the patient's weight loss goals, pre-existing comorbidities, willingness to comply with the required dietary and lifestyle changes, and the patient's preference are also important factors to consider when making the decision as to the choice of an optimal surgical procedure for each patient.[37]

In our model, older age, male sex, and higher baseline weight were positively associated with post-LAGB WL and EWL over time. Higher baseline excess weight was negatively associated with EWL after surgery. Given the known limitations of %EWL as an outcome measure for weight loss (e.g., the heavier the patient, the smaller the %EWL) [38], the effect of baseline WL (in the %WL model) provides a more clinically meaningful estimate. Older patients lost more weight than younger patients, and men lost a higher percentage of excess weight than women (although there was no difference between men and women in percentage weight loss).

The model used here is a novel approach to providing a more accurate assessment of post-bariatric surgery weight loss in the real-world clinical setting where the compliance of individual patients to recommended follow-up visits can be highly variable. The traditional approach to evaluating post-surgical weight loss parameters is based on simple arithmetic means, which frequently requires interpolation to “best-fit” the actual patient visits to pre-determined study time points and may omit patient data points that do not fit within specified “visit windows”, introducing additional sources of bias into the calculation of the population means. The advantages of the model used here are that it incorporates all data points for each patient, requires no data reduction or imputation, and uses the weight loss outcomes achieved by each individual patient to generate a statistically more accurate estimate for the mean weight loss outcome for the overall population.

Further studies are needed to determine whether the pre-operative patient characteristics used in this model can be used to provide a clinically relevant prediction of the post-operative weight loss in an individual patient over time. Until further data are available, these pre-operative factors may be of use to the clinician for counseling patients on expected outcomes based on the baseline characteristics of the individual patient.

Adverse events were relatively low in the overall patient population, with 238 of 2,815 patients (8.5 %) experiencing a total of 260 adverse events during a mean follow-up of 21 months and a follow-up of 5 years in 74 patients. The most frequent adverse event in our population was PPD (band slippage or pouch dilatation), which occurred in 118 patients (4.2 %). The only other adverse events that occurred in more than 1 % of patients were tubing/access port problems (*n* = 35; 1.2 %) and band explantation (*n* = 35; 1.2 %). The rates of adverse events observed in this study are on the low end of those reported in the literature,[15, 18], although others have also reported similarly low rates [17]. Adverse events may be influenced by surgical technique (e.g., pars flaccida, which was used exclusively in the present study, vs. perigastric) [39], the type of band used, frequency of follow-up (but not necessarily adjustments), and surgeon experience [11]. Nine (0.3 %) patients in this study population died over the 5 years encompassed by the analysis. In the eight patients for whom causes of death could be ascertained, none was believed to be related to LAGB treatment.

An additional consideration influencing the rate of long-term LAGB complications pertains to the differences in healthcare systems in various countries. In Canada, there may be a greater tendency to revise the band or port rather than explant the device because wait times for revision procedures such as gastric bypass surgery may be as long as 3 years. In contrast, revision surgery to remove the band and perform a second procedure may be more readily available in the USA and Europe. This may lead to higher rates of explantation or secondary procedures rather than band retention with revision. Other local and regional factors could influence the frequency of selected long-term complications and their management.

Additionally, the percentage of patients LFU in this study was low (19 %) considering its long-term nature. We recognize that patients who are lost to follow-up may not be captured in the reporting of adverse events, and it is possible that some patients who underwent band explantation did not report this to our clinic. However, the nature of the Canadian healthcare system and the limited access to revision surgery would likely encourage patients with adverse events to follow up with the clinic at which the band was implanted. LFU patients are not captured in the weight loss data beyond their last follow-up time point, and therefore we cannot make any assumptions as to whether weight loss was maintained, increased, or decreased over time. This limitation is common to all long-term studies of bariatric surgeries, many of which do not report the rates of LFU, thereby affecting the generalizability of the data presented.

Overall, the present study adds to a growing literature establishing the medium- to long-term stability of weight loss with LAGB. Notably, the 5-year weight loss experience in the outpatient setting reported here is consistent with other long-term studies demonstrating clinically meaningful and sustained weight loss outcomes with LAGB.[3] Consequently, the maintenance of weight loss, combined with the low rates of adverse events and the ability to routinely perform the procedure in an outpatient setting, makes LAGB one of several effective surgical options for obese patients. As all bariatric surgery procedures require long-term behavioral and lifestyle changes by the patient, it is important that the clinician consider both the safety and efficacy of the surgery, along with patient preferences, to determine the most appropriate plan for the individual patient.
